# Decrease in the Photosynthetic Performance of Temperate Grassland Species Does Not Lead to a Decline in the Gross Primary Production of the Ecosystem

**DOI:** 10.3389/fpls.2018.00067

**Published:** 2018-02-05

**Authors:** Anthony Digrado, Louis G. de la Motte, Aurélie Bachy, Ahsan Mozaffar, Niels Schoon, Filippo Bussotti, Crist Amelynck, Anne-Catherine Dalcq, Marie-Laure Fauconnier, Marc Aubinet, Bernard Heinesch, Patrick du Jardin, Pierre Delaplace

**Affiliations:** ^1^Plant Biology Laboratory, AGRO-BIO-CHEM, University of Liège-Gembloux Agro-Bio Tech, Gembloux, Belgium; ^2^Biosystems Dynamics and Exchanges, TERRA, University of Liège-Gembloux Agro-Bio Tech, Gembloux, Belgium; ^3^Royal Belgian Institute for Space Aeronomy, Uccle, Belgium; ^4^Department of Agri-Food Production and Environmental Science, University of Florence, Florence, Italy; ^5^Department of Analytical Chemistry, Ghent University, Ghent, Belgium; ^6^Modelling and Development Unit, AGRO-BIO-CHEM, University of Liège-Gembloux Agro-Bio Tech, Gembloux, Belgium; ^7^Agro-Bio Systems Chemistry, TERRA, University of Liège-Gembloux Agro-Bio Tech, Gembloux, Belgium

**Keywords:** carbon fluxes, chlorophyll fluorescence, eddy covariance, grassland, GPP, JIP-test, respiration

## Abstract

Plants, under stressful conditions, can proceed to photosynthetic adjustments in order to acclimatize and alleviate the detrimental impacts on the photosynthetic apparatus. However, it is currently unclear how adjustment of photosynthetic processes under environmental constraints by plants influences CO_2_ gas exchange at the ecosystem-scale. Over a 2-year period, photosynthetic performance of a temperate grassland ecosystem was characterized by conducting frequent chlorophyll fluorescence (ChlF) measurements on three primary grassland species (*Lolium perenne* L., *Taraxacum* sp., and *Trifolium repens* L.). Ecosystem photosynthetic performance was estimated from measurements performed on the three dominant grassland species weighed based on their relative abundance. In addition, monitoring CO_2_ fluxes was performed by eddy covariance. The highest decrease in photosynthetic performance was detected in summer, when environmental constraints were combined. Dicot species (*Taraxacum* sp. and *T. repens*) presented the strongest capacity to up-regulate PSI and exhibited the highest electron transport efficiency under stressful environmental conditions compared with *L. perenne*. The decline in ecosystem photosynthetic performance did not lead to a reduction in gross primary productivity, likely because increased light energy was available under these conditions. The carbon amounts fixed at light saturation were not influenced by alterations in photosynthetic processes, suggesting photosynthesis was not impaired. Decreased photosynthetic performance was associated with high respiration flux, but both were influenced by temperature. Our study revealed variation in photosynthetic performance of a grassland ecosystem responded to environmental constraints, but alterations in photosynthetic processes appeared to exhibit a negligible influence on ecosystem CO_2_ fluxes.

## Introduction

Continuous exposure to environmental constraints can negatively affect plant growth and cause yield loss in agricultural crop plants. In particular, environment affects photosynthesis. Photosynthesis and the photosynthetic apparatus can be respectively altered and potentially damaged by elevated air temperatures, high light levels, and drought (Bussotti et al., 2007; Bertolde et al., 2012; Goh et al., 2012; Ashraf and Harris, 2013). Plants, however, can proceed to photosynthetic adjustments in order to acclimatize under stressful conditions and alleviate the detrimental impacts on the photosynthetic apparatus. For instance, under high light, plants promote the dissipation of excess energy within the light harvesting complex to decrease photosystem II (PSII) excitation (Goh et al., 2012; Jahns and Holzwarth, 2012). Plants can also protect PSII by adjusting the electron transport rate within the PSII reaction center (RC) or passing electrons to alternative electron acceptors (Derks et al., 2015). Cyclic electron flow around photosystem I (PSI) can be triggered to alleviate photoinhibition, as it contributes to the generation of an acidic lumen, which promotes energy dissipation via heat through the xanthophyll cycle (Takahashi et al., 2009). Oxidative stress can also result from an imbalance between dark and light-phases of photosynthesis under stressful environmental conditions. In such cases, ATP and NADPH accumulate, but are not converted to ADP and NADP^+^ to feed the primary reactions (e.g., light phase). Photorespiration, by using ATP and NADPH energy, enables acceptor regeneration for photosynthetic primary reactions and prevents reactive oxygen species (ROS) production (Voss et al., 2013).

Perturbation of photosynthetic processes can impact plant photosynthetic performance, which is defined here as the efficiency in which a photon can be absorbed and used to produce chemical energy. Chlorophyll *a* fluorescence (ChlF) analysis was employed in several studies to investigate the physiological aspects of photosynthesis and characterize plant photosynthetic performance (Murchie and Lawson, 2013; Guidi and Calatayud, 2014). Measure of the fast fluorescent transient (i.e., the OJIP curve) of dark-adapted leaves using a plant efficiency analyser gives information on the “potential” plant photosynthetic performance, whereas a pulse-amplitude modulated fluorimeter provides measures of the actual plant photosynthetic efficiency (Kalaji et al., 2017). Analysis of the OJIP curve derived from a plant efficiency analyser fluorimeter provides useful information related to the photosynthetic apparatus status and functioning (Maxwell and Johnson, 2000). Studies reported ChlF transient analysis using the JIP-test a very useful tool to investigate the photosynthetic apparatus response and adaptive ability to a wide range of stressors (Bussotti et al., 2007, 2010; Redillas et al., 2011; Brestic et al., 2012). Its utilization in ecological studies also demonstrated its relevance in assessing different taxa for photosynthetic properties and characterizing taxon adaptive photosynthetic strategies (Pollastrini et al., 2016a). The JIP-test applies energy fluxes in biomembrane theory to calculate several phenomenological and biophysical parameters that characterize PSII behavior and therefore photosynthetic performance (Strasser et al., 2000). PSI activity was later described using new parameters (Tsimilli-Michael and Strasser, 2008; Smit et al., 2009), which enabled the study of energy fluxes from the plastoquinone pool to the PSI acceptor side. Many studies have contributed to increase the empirical knowledge on the possible association between the shape of the fluorescence transient and the physiological status of the studied sample (Kalaji et al., 2016).

The relationship between the fast fluorescent transient and gas exchange, however, is not straightforward and is still largely debated (Kalaji et al., 2017). While reduced PSII efficiency can be associated with a decreased carbon assimilation (van Heerden et al., 2003; Bertolde et al., 2012), reduction in net photosynthesis are typically related to stomatal limitations (Bollig and Feller, 2014), and/or inactivation of the rubisco enzyme (Feller, 2016). Indeed, a decline in photosynthetic activity can be observed during moderate heat stress without impairment of PSII (Stroch et al., 2010; Pettigrew, 2016) as the entire photosynthetic process is affected at lower temperature than its PSII component alone (Janka et al., 2013). Furthermore, Lee et al. (1999) reported PSII complexes were sometimes observed in excess in leaves and the photosynthetic apparatus is able to suffer considerable damages before a reduction in net photosynthesis occurs. Despite these observations, different photosynthetic responses among species under equal environmental constraints were reported, likely from variable abilities to efficiently use photon energy under stressful conditions (Gravano et al., 2004; Jedmowski et al., 2014; Kataria and Guruprasad, 2015). Differences in photosynthetic component sensitivity to abiotic stress (Oukarroum et al., 2016) also explained varied photosynthetic response to environmental constraints. As a consequence, plants living under different climatic conditions expressed variation in their photosynthetic performance and sensitivity to environmental factors due to local adaptation (Ciccarelli et al., 2016; Pollastrini et al., 2016a,b). This emphasizes the importance to better characterize the photosynthetic functionality of different ecosystems in varied climatic conditions to circumvent climate change impacts on photosynthesis.

Here, we addressed the following questions: (i) do different species and/or taxonomic groups display different response mechanisms; and (ii) do variations in the photosynthetic performance in a grassland ecosystem affect CO_2_ fluxes at the ecosystem level. In a previous study, we detected important seasonal variation in the photosynthetic performance of the perennial ryegrass (*Lolium perenne* L.) in a temperate grassland due to the influence of combined environmental constraints (Digrado et al., 2017). However, we did not compare *L. perenne* response to those of other grassland species neither analyse if variations in photosynthetic performance were associated with changes in carbon assimilation by the ecosystem. Indeed, it is likely that grassland species differing in various anatomic aspect (leaf shape, root system…) might express contrasted photosynthetic responses. In such case, it is reasonable to assume that the photosynthetic performance at the ecosystem level must be influenced by its different components (i.e., photosynthetic response at leaf scale for the different species). Based on the current literature, we hypothesize that different species may be characterized by a different photosynthetic performance response explained either by a different acclimatization strategy, stress perception or metabolic response. We also hypothesize that increase in energy dissipation mechanisms at the ecosystem level may be associated with a decrease or a plateau in carbon assimilation as proportionally less photon energy is used for photochemistry. To test our hypothesis, we measured over two growing seasons in field by ChlF the photosynthetic performance response of *L. perenne* and two other important grassland species. ChlF parameters derived from the JIP-test were used to determine which mechanisms triggered the photosynthetic apparatus under stressful environmental conditions and if they differed among species. We also estimated ecosystem level photosynthetic performance by extrapolating leaf-scale measurements and addressed whether an alteration in ecosystem photosynthetic performance affected ecosystem CO_2_ fluxes measured using the eddy covariance technique.

## Materials and methods

### Field site

The study was conducted within the CROSTVOC project (CROp Stress Volatile Organic Compounds: http://hdl.handle.net/2268/178952) framework. All measurements were performed at the Dorinne Terrestrial Observatory in Belgium (50°18′44″N 4°58′07″E). The climate at the site is temperate oceanic. The study area supports permanent grassland, which covers 4.22 ha, and the relief is dominated by a large colluvial depression oriented south-west/north-east. The depression lies on a loamy plateau with a calcareous and/or clay substrate. Altitudes range from 240 m (north-east) to 272 m (south). The paddock was converted to permanent grassland at least 50 years before the start of this study and was intensively grazed by cattle, with the application of cattle slurry and manure. Botanical diversity was evaluated on 24 quadrats (0.5 × 0.5 m) during the months of September 2010 and June 2011. Plant communities were composed of 13 grass species [*Agrostis stolonifera* L., *Alopecurus geniculatus* L., *Bromus hordeaceus* L., *Cynosurus cristatus* L., *Dactylis glomerata* L., *Elymus repens* (L.) Gould, *Festuca pratensis* Huds., *Holcus lanatus* L., *Lolium multiflorum* Lam., *L. perenne, Poa annua* L., *Poa pratensis* L., and *Poa trivialis* L.], one N-fixing dicot (*Trifolium repens* L.), and seven non-N-fixing dicots [*Capsella bursa-pastoris* (L.) Medik., *Carduus* sp. L., *Matricaria discoidea* DC., *Plantago major* L., *Ranunculus repens* L., *Stellaria media* (L.) Vill., *Taraxacum* sp.]. The dominant species were *L. perenne, Taraxacum* sp. and *T. repens* (Supplementary Table 1).

### Micro-meteorological data

Grassland micro-meteorological data, including photosynthetic photon flux density (PPFD) (SKP215, Skye Instruments, Llandrindod Wells, UK), air temperature (*T*_air_), air relative humidity (RH) (RHT2nl, Delta-T Devices Ltd., Burwell, Cambridge, UK) at 2.62 m above soil level, soil moisture (SM) (CS616, Campbell Scientific Inc., Logan, UT, USA), and soil temperature (*T*_soil_) (PT 1000, Campbell Scientific Inc., Logan, UT, USA) at 2 cm depth were recorded every 30 min throughout the measurement period. Vapor pressure deficit (VPD) was calculated from *T*_air_ and RH measurements.

### CO_2_ flux measurements

CO_2_ flux measurements and computation procedures were performed as described by Gourlez de la Motte et al. (2016). High frequency flux losses were corrected following Mamadou et al. (2016). CO_2_ flux measurements were conducted using the eddy covariance technique and a three-dimensional sonic anemometer (CSAT3, Campbell Scientific Ltd., UK) coupled with a fast CO_2_-H_2_O non-dispersive infrared gas analyser (IRGA) (LI-7000, LI-COR Inc., Lincoln, NE, USA) to measure CO_2_ fluxes. Air was drawn into the IRGA through a tube (6.4 m long; inner diameter 4 mm) by a pump (NO22 AN18, KNF Neuberger, D) with a 12 l min^−1^ flow at a height of 2.6 m above ground. Data were sampled at a rate of 10 Hz. Zero and span calibrations were performed for CO_2_ approximately once per month. Pure nitrogen (Alphagaz 1, Air Liquide, Liege, Belgium) was used for the zero and 350 μmol CO_2_ mol^−1^ mixture (Chrystal mixture, Air Liquide, Liege, Belgium) for the span. Net ecosystem CO_2_ exchange (NEE) from half-hourly eddy covariance measurements was partitioned into gross primary production (GPP) and total ecosystem respiration (R_eco_) according to the method described by Reichstein et al. (2005).

The GPP response to PPFD for days where ChlF measurements were performed was fit by a Mitscherlich equation (Dagnelie, 2013) where the quantum light efficiency *a* (i.e., the initial slope of the curve) and GPP_max_ (i.e., the asymptotic value of GPP for PPFD→ ∞) were deduced using R software version 3.3.0 (R Development Core Team, 2012). Only measurements with PPFD-values above 50 μmol m^−2^ s^−1^ were applied during the procedure. The equation was as follows:

$$\begin{array}{l} GPP\;\left(PPFD\right)=GPP_{max}\;\times \;\left[1-\;exp^{\left(-\;\frac{\alpha \;\times \;PPFD}{GPP_{max}}\;\right)}\right] \end{array}$$

This equation was used to calculate GPP_1500_ (i.e., GPP at PPFD = 1,500 μmol m^−2^ s ^−1^) to estimate GPP at light saturation. This parameter was used in this study rather than GPP_max_ because the latter can lead to GPP overestimation at light saturation. Conclusions of this study were not dependent on this choice, as similar results were obtained applying both parameters.

The response of *R*_eco_ to *T*_soil_ for days when ChlF measurements were conducted was fit based on the Lloyd and Taylor (1994) equation where the activation energy *E*_0_ (i.e., respiration sensitivity to temperature) and *R*_10_ (i.e., dark respiration normalized at 10°C) were deduced from R software version 3.3.0. The equation was as follows:

$$\begin{array}{l} R_{eco}\;\left(T_{soil}\right)=R_{10}\times exp\;\left[E_{0}\times \left(\frac{1}{T_{ref}+46.02}-\frac{1}{T_{soil}+46.02}\right)\right] \end{array}$$

The reference temperature (*T*_ref_) was set at 10°C.

Because, respiratory activity is extrapolated from night measurements, CO_2_ fluxes estimated at day may have been biased because of several mechanisms related to plant physiology that were not taken into account in this method. The omission of the inhibition of plant mitochondrial respiration by light (Atkin et al., 1997), for instance, may have led to an overestimation of *R*_eco_ at day (Heskel et al., 2013; Wehr et al., 2016). In contrast, the omission of photorespiratory activity may have led to an underestimation of *R*_eco_ (Wohlfahrt and Gu, 2015), especially during hot days when photorespiration is high due to the increasing specificity of Rubisco with O_2_ at high temperature (Sage, 2013). This have consequences on GPP estimation since it is derived from NEE and *R*_eco_. These issues are well known by the eddy covariance researcher community and it is difficult to quantify the contribution of each potential biases in our measurements. By using an adapted “big-leaf photosynthesis model,” a study was able to assess the bias in CO_2_ fluxes measured by eddy covariance in response to light (Wohlfahrt and Gu, 2015). They showed that *R*_eco_ was overestimated at low light (ca. <800 μmol m^−2^ s^−1^) because the mitochondrial inhibition bias overweighed photorespiration in these conditions. In contrast, *R*_eco_ was underestimated at high light (ca. >800 μmol m^−2^ s^−1^) because photorespiration became stronger. They also showed that, over a daily cycle, GPP was more representative of the “true photosynthesis” (i.e., gross photosynthesis, carboxylation) rather than the “apparent photosynthesis” (i.e., true photosynthesis minus photorespiration) and only overestimated “true photosynthesis” by 3% on average. This was acknowledged in the interpretations of our results.

### Analysis of the fluorescence transient using the JIP test

Grassland ChlF emission measures were conducted on *L. perenne, Taraxacum* sp., and *T. repens* at three plots (each 30 × 5 m) from June to October 2014 and May to October 2015, using a HandyPEA fluorimeter (Hansatech Instruments, Pentney, Norfolk, UK). Cows were allowed to graze between measurement periods. Measurements were performed in each monitored plot 4x day^−1^ at 11:00, 13:00, 15:00, and 17:00 h (local time zone). The number of replicates for each plot and time period was seven in 2014 and eight in 2015. Measurements were performed on non-senescent mature leaves. Prior to each measurement, leaves were dark-adapted with leaf clips for 30 min. The leaf clips applied to *L. perenne* were modified to fit the width of leaves by reducing the measurement surface by half using black vinyl electrical tape, following the manufacturer's recommendations. The dark-acclimated leaf surfaces were then exposed to red light with a flux density of 3,000 μmol m^−2^ s^−1^ for 1 s, which was provided with an array of three light-emitting diodes (peak wavelength at 650 nm). During the 1 s-long excitation with the red light of the sample, the induced fluorescence signal was recorded every 10 μs from 10 to 300 μs, then every 100 μs till 3 ms, then every 1 ms till 30 ms, then every 10 ms till 300 ms, and finally every 100 ms till 1 s.

Fluorescence emissions measured at 50 μs (*F*_50_, step O), 300 μs (*F*_300_, step K), 2 ms (step J), 30 ms (step I), and maximum (*F*_M_, step P) were used to characterize fluorescence emission transients and determine several parameters describing photosynthetic activity based on the JIP test (Strasser et al., 2000, 2004). Table 1 summarizes the ChlF parameters used in this study. When a ChlF parameter showed an aberrant value (i.e., infinite), all parameters derived from this specific measurement were discarded. This represented <0.16% of the dataset.

**Table 1:** Summary of parameters and formulas used for the analysis of the fast fluorescence transient OJIP.

**Parameters**	**Equations**	**Description**
**TECHNICAL FLUORESCENCE PARAMETERS**		
*F_*t*_*		Fluorescence intensity at the time *t*.
*F*_50_		Fluorescence intensity at 50 μs (O-step).
*F*_300_		Fluorescence intensity at 300 μs (K-step).
*F*_J_		Fluorescence intensity at 2 ms (J-step).
*F*_I_		Fluorescence intensity at 30 ms (I-step).
*F*_M_		Maximum fluorescence intensity (P-step).
*F*_V_	*F*_m_ – *F*_50_	Maximum variable fluorescence.
*F*_V_/*F*_M_ (= ϕ_P0_)	1 – (*F*_50_/*F*_M_)	Maximum quantum yield of PSII of a dark adapted leaf. Expresses the probability that an absorbed photon will be trapped by the PSII reaction center.
**JIP-TEST DERIVED PARAMETERS**		
*M*_0_	4.[(*F*_300−_*F*_50_) / (*F*_M_ – *F*_50_)]	Approximate initial slope of the fluorescence transient.
*V_*t*_*	(*F_*t*_*_−_*F*_50_)/(*F*_M_ – *F*_50_)	Relative variable fluorescence at the time *t*.
*V*_J_	(*F*_J−_*F*_50_) / (*F*_M_ – *F*_50_)	Relative variable fluorescence at 2 ms (J-step).
*V*_I_	(*F*_I−_*F*_50_) / (*F*_M_ – *F*_50_)	Relative variable fluorescence at 30 ms (I-step).
RC/ABS	ϕ_P0_ (*V*_J_ / *M*_0_)	Q_A_-reducing reaction centers (RC) per PSII antenna Chl.
Ψ_E0_ (= J phase)	1 – *V*_J_	The efficiency with which a photon trapped by the PSII RC moves an electron into the electron transport chain beyond Q_A_.
Δ*V*_IP_ (= I–P phase)	1 – *V*_I_	The efficiency with which a photon trapped by the PSII RC moves an electron into the electron transport chain beyond PSI to reduce the final acceptors of the electron transport chain, i.e., ferredoxin and NADP^+^.
PI_ABS_	(RC/ABS) [ϕ_P0_ / (1 – ϕ_P0_)] [Ψ_E0_ / (1 – Ψ_E0_)]	Performance index (potential) on absorption basis for energy conservation from photons absorbed by PSII to the reduction of intersystem electron acceptors.

*The nomenclature used is in agreement with the work of Strasser et al. (2000, 2004)*.

### Statistical analyses

Three groups of comparable meteorological conditions were defined by clustering (Ward's method based on the Euclidian distance). Clustering was performed on the coordinates of the two first principal components of a principal component analysis (PCA) (result of the PCA-clustering available in Supplementary Figure 1A). All meteorological parameters (PPFD, *T*_air_, VPD, SM, RH, and *T*_soil_) were entered as variables in the PCA. The influence of monitored plots on ChlF response was examined using a General Linear Model (GLM) type III. The assigned cluster number previously identified for each time period of measurement were used as a meteorological factor in the GLM analysis. The monitored plots were used in the GLM as a block factor and was considered as random factor. Plot influence was not found significant; therefore ChlF parameter values were averaged for the three species without consideration of the different plots.

Ecosystem photosynthetic performance was approximated from measurements performed on the three dominant grassland species. In order to achieve this, ChlF parameters from the three species were weighed based on the relative abundance of their respective group (i.e., grass, N-fixing dicot, and non-N-fixing dicot) to extrapolate ChlF parameters for the entire pasture/grassland ecosystem. Relative abundance values from the last survey were used (Supplementary Table 1). Three groups of contrasting ecosystem photosynthetic performance based on ecosystem ChlF parameters were then defined using the same methodological approach employed in the meteorological groups (result of the PCA-clustering available in Supplementary Figure 1B). Ecosystem ChlF parameters (*F*_V_/*F*_M_, PI_ABS_, Ψ_E0_, and Δ*V*_IP_) were entered as variables in this second PCA followed by clustering, with the exception of the raw fluorescence values *F*_50_ and *F*_M_ which were used to calculate ChlF parameters. The behavior of the three grassland species within these groups was analyzed. A type III GLM and Tukey's Honest Significant Difference test (Tukey HSD) were used to classify mean ChlF parameter values at species and ecosystem levels, meteorological mean parameter values and CO_2_ fluxes among ChlF clusters. Prior to statistical analyses, a correction factor based on the reduction ratio surface measurements of *L. perenne* leaf clips was applied to *F*_50_ and *F*_M_-values to enable the monocot comparisons with those measured on dicot species. ChlF parameters (except for PI_ABS_ due to the presence of negative values) and CO_2_ fluxes were square root-transformed for type III GLM analyses to improve normality and homogeneity of variances. Photosynthetic processes influence on daily variation in ecosystem respiration and grassland capacity to fix carbon at light saturation were tested by simple linear regressions between the daily amplitude variability in ecosystem ChlF parameters and *R*_10_ and GPP_1500_.

Relationships among ChlF-based photosynthetic responses, meteorological conditions, and CO_2_ fluxes were explored using Canonical Correlation Analysis (CCA). This multivariate statistical test serves to identify linear combinations among random variables between two datasets (e.g., ChlF and meteorological data) in order to maximize their correlation. The two new sets of canonical variates, constructed based on the original datasets, determine a pair of canonical variates with a maximized simple correlation. Each set of constructed canonical variates has a variance = 1 and are uncorrelated with other constructed variates. The correlation relevance between the two groups of datasets is tested by the significance of the correlation between the pairs of canonical variates. Significance in at least one canonical pair means the two analyzed datasets are not independent and enable the associations among the different variables. Compared with the Pearson correlation test, CCA explores interdependence within a set of variables. Approach of the canonical correlation is very similar to PCA as axis are a linear combination of data. However, in contrast to PCA where linear combination maximizes the variance on the first axis, CCA maximizes the correlation between two datasets. Three CCA analysis were performed by combining ChlF and meteorological data; meteorological and CO_2_ fluxes data; and ChlF and CO_2_ fluxes data. An alternative representation of the relationship between ChlF response and meteorological conditions is available in Supplementary Material where clusters of contrasted meteorological condition were represented along clusters of contrasted ecosystem photosynthetic performance in the PCA environment computed based on ChlF data (Supplementary Figure 1C).

All analyses were performed using the software Minitab® version 17.1.0 (State College, Pennsylvania, USA) and R software version 3.3.0 with the following R package: “FactoMineR” (Husson et al., 2016), “Vegan” (Oksanen et al., 2012), “CCP” (Menzel, 2015), “Car” (Fox et al., 2016), and “Relaimpo” (Groemping and Matthias, 2013).

## Results

### Environmental conditions

**Figure 1:**
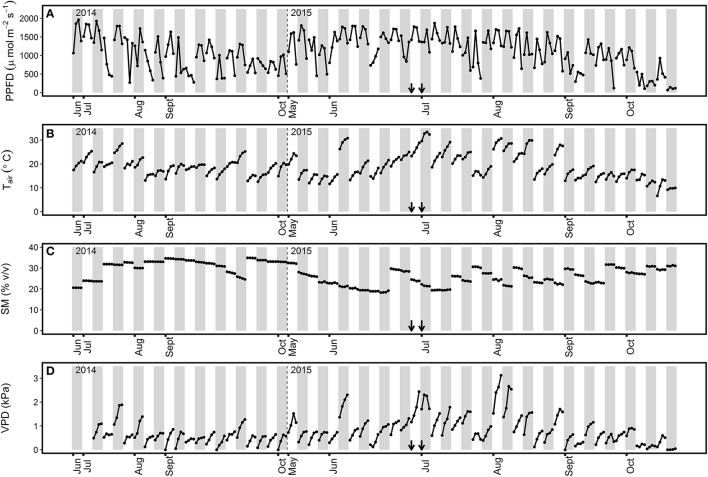
Environmental conditions encountered during chlorophyll fluorescence measurements in the 2014 and 2015 study periods. Values at 11, 13, 15, and 17 h for each day of chlorophyll fluorescence measurements are represented for **(A)** PPFD, photosynthetic photon flux density; **(B)** T_air_, air temperature; **(C)** SM, soil moisture at a depth of 5 cm; **(D)** VPD, vapor pressure deficit. Gray bars separate the different days of measurements. The arrows indicate the first and third day of the heat wave. Modified from Digrado et al. (2017).

The highest solar irradiance values were detected during summer (i.e., May to August) for both years, with midday PPFD-values above 1,500 μmol m^−2^ s^−1^ (Figure 1A). This period was also characterized by the highest midday *T*_air_ and VPD-values, which exceeded 30°C and 2.0 kPa, respectively (Figures 1B,D). Autumn (i.e., September to October) was characterized by PPFD, *T*_air_, and VPD-values below 1,500 μmol m^−2^ s^−1^, 25°C, and 1 kPa, respectively, for most ChlF measurement days. Three specific meteorological events were observed in the grassland during the second year of the study. A heat wave from 30 June to 5 July 2015 (Figure 1A) characterized by six consecutive days with maximum *T*_air_ between 28.9 and 34°C, and two dry spells with ~20% SM during the months of June and July 2015 (Figure 1C).

### Grassland ChlF parameters evolution and influence of environmental conditions

**Figure 2:**
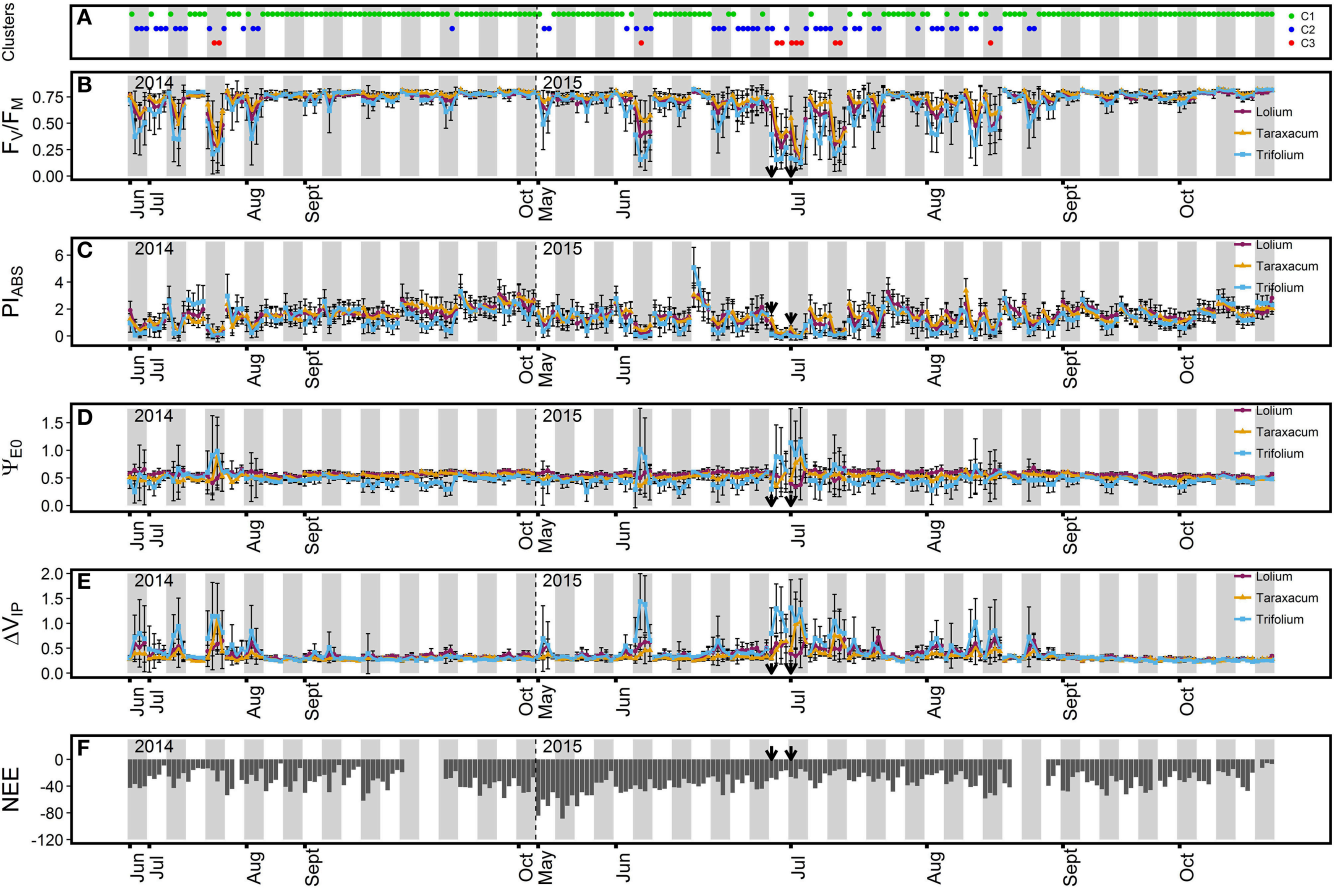
Time course of ChlF parameters (**B**, F_V_/F_M_; **C**, PI_ABS_, **D**, Ψ_E0_; and **E**, Δ*V*_IP_) for the three grassland species and net ecosystem CO_2_ exchange (**F**, NEE, μmol CO_2_ m^−2^ s^−1^) during the two measurement campaign in the grassland. For each measured day, the ChlF parameter average value (*n* = 21 or 24) ± SD for each of the four measurement time periods (11:00, 13:00, 15:00, and 17:00 h) is represented for the three grassland species (purple, *L. perenne*; orange, *Taraxacum* sp.; light blue, *T. repens*). **(A)** ChlF cluster (green, C1; dark blue, C2; red, C3) the group has been assigned during each time period by the Principal Component Analysis (PCA)-clustering. Gray bars separate the different days of measurements. The arrows indicate the first and third day of a heat wave. A focus on ChlF parameters and NEE variations during the heat wave period is available in Supplementary Figure 2.

Grassland ChlF parameters exhibited diurnal variation and showed a pattern consistent with PPFD (Figures 1A, 2). Indeed, while other environmental parameters such as *T*_air_ and VDP were still high at the end of the day, ChlF parameters usually went back to a value similar to that of the first measurement performed, following the diurnal pattern of PPFD. Variation in ChlF parameters for the three grassland species was highest in summer, where the greatest diurnal increases/decreases were measured around midday, when high PPFD-values were measured with high *T*_air_ and VPD. During the midday period, frequent declines in absorbed photon energy use efficiency, expressed as a strong decrease in maximum quantum yield of primary photochemistry (*F*_V_/*F*_M_) and performance index (PI_ABS_) were observed for the three grassland species. These declines were characterized by decreased *F*_V_/*F*_M_- and PI_ABS_-values as low as 0.12 and < 0.01, respectively. In contrast, a stable *F*_V_/*F*_M_-value of ~0.76 was measured in autumn for the three grassland species, indicating high PSII performance during this period. During the summer sampling period, observed midday decreases in *F*_V_/*F*_M_ were accompanied by increases in the efficiency to reduce the end electron acceptor (Δ*V*_IP_) for the three measured grassland species (Figure 2E). More stable Δ*V*_IP_-values of ~0.31 were observed in autumn. Reduced variation in electron transport efficiency beyond Q_A_ (Ψ_E0_) (Figure 2B) was observed, with stable values of ~0.53 for Ψ_E0_. Changes in Ψ_E0_ were only observed during episodes with high increased Δ*V*_IP_. During these episodes, the three grassland species presented the following contrasting Ψ_E0_ response: strong augmentation in Ψ_E0_ were observed in *Taraxacum* sp. and *T. repens*, whereas *L. perenne* showed reduced variation or even decreased Ψ_E0_.

Three groups of contrasted ecosystem photosynthetic performance were determined by PCA-clustering (Table 2). The first group (C1) was characterized by a high capacity to employ photon energy, illustrated by high *F*_V_/*F*_M_- and PI_ABS_-values for the ecosystem and the three grassland species. C1 cluster response was characterized as dominant in autumn and was detected in 171 observed time periods (Figure 2A). *L. perenne* and *Taraxacum* sp. exhibited the highest PI_ABS_ values in the C1 group. In contrast, the third group (C3) was characterized by the lowest *F*_V_/*F*_M_ and PI_ABS_-values for the grassland ecosystem and the three species, demonstrating low photosynthetic performance. The photosynthetic response characterized by this group was detected in 11 observed time periods and the responses were only detected in summer, particularly at midday (Figure 2A). *Taraxacum* sp. presented the highest *F*_V_/*F*_M_-values in this group, whereas the lowest values were observed in *T. repens*. The second group (C2) showed intermediate *F*_V_/*F*_M_- and PI_ABS_-values, where *Taraxacum* sp. exhibited the highest *F*_V_/*F*_M_-values in C2. Photosynthetic response characterized by this cluster was observed throughout the season in 54 observed time periods (Figure 2A). These results indicated that the grassland ecosystem was more efficient in photon energy use for photochemistry in autumn. Increased initial fluorescence (*F*_50_) and decreased maximum fluorescence yield (*F*_M_) were responsible for decreased *F*_V_/*F*_M_-in clusters C2 and C3 (Table 2). Only moderate Ψ_E0_ changes were observed in the three species between the C1 and C2 clusters. However, results indicated differences in electron transport efficiency between dicot and monocot species beyond Q_A_ in the C3 cluster. Indeed, dicot species exhibited either a stable (non-significant Ψ_E0_ increase for *Taraxacum* sp. even though high values were recorded in July 2014 and July 2015) or an increased value (+86% Ψ_E0_ increase for *T. repens*) of Ψ_E0_ whereas a decrease in Ψ_E0_ was observed for *L. perenne* (−19%). *F*_V_/*F*_M_-and PI_ABS_-decreased values were accompanied by increases in Δ*V*_IP_ for dicot species in clusters C2 and C3. *L. perenne*, however, did not exhibit a significant change in Δ*V*_IP_ between the cluster C2 and C3 despite changes in environmental conditions (Table 2). The photosynthetic ecosystem response based on ChlF parameters was similar to *L. perenne* as the contribution of grass species to the ecosystem response was estimated at 88.6%.

**Table 2:** Description of the three clusters of photosynthetic performance (PP) based on ChlF measurements (C1, C2, and C3) for each grassland species and the ecosystem, and the prevailing micrometeorological conditions.

			**Average in the Different Clusters and Associated Relative Change**				
			**C1**	**C2**	**Δ%**	**C3**	**Δ%**
Species PP	Lolium	*F*_V_/*F*_M_	0.755^A^	0.610^C^	−19	0.322^F^	−57
		PI_ABS_	1.807^A^	0.894^C^	−51	0.150^D^	−92
		Ψ_E0_	0.565^C^	0.608^B^	+8	0.458^EF^	−19
		Δ*V*_IP_	0.324^E^	0.516^C^	+59	0.497^C^	+54
		*F*_50_	459^F^	558^D^	+21	791^B^	+72
		*F*_M_	1895^B^	1530^D^	−19	1192^E^	−37
	Taraxacum	*F*_V_/*F*_M_	0.768^A^	0.662^B^	−14	0.398^E^	−48
		PI_ABS_	1.736^AB^	1.052^C^	−39	0.269^D^	−85
		Ψ_E0_	0.531^D^	0.520^DE^	−2	0.568^BCD^	+7
		Δ*V*_IP_	0.294^F^	0.384^D^	+31	0.687^B^	+134
		*F*_50_	519^E^	607^C^	+17	856^B^	+65
		*F*_M_	2262^A^	1940^B^	−14	1517^D^	−33
	Trifolium	*F*_V_/*F*_M_	0.746^A^	0.503^D^	−33	0.206^G^	−72
		PI_ABS_	1.592^B^	0.541^D^	−66	0.072^D^	−95
		Ψ_E0_	0.470^F^	0.472^F^	0	0.618^A^	+89
		Δ*V*_IP_	0.312^EF^	0.618^B^	+98	1.139^A^	+264
		*F*_50_	566^D^	821^B^	+45	1178^A^	+108
		*F*_M_	2277^A^	1805^C^	−21	1500^D^	−34
Ecosystem PP		*F*_V_/*F*_M_	0.755^A^	0.607^B^	−20	0.320^C^	−58
		PI_ABS_	1.791^A^	0.884^B^	−51	0.153^C^	−91
		Ψ_E0_	0.558^B^	0.595^A^	+7	0.489^C^	−12
		Δ*V*_IP_	0.322^B^	0.514^A^	+60	0.544^A^	+62
		*F*_50_	469^C^	575^B^	+23	816^A^	+74
		*F*_M_	1938^A^	1568^B^	−19	1228^C^	−37
Meteorological conditions		PPFD	917^B^	1463^A^	+59	1639^A^	+79
		*T*_air_	17.06^C^	23.84^B^	+40	28.27^A^	+66
		VPD	0.59^B^	1.34^A^	+129	1.67^A^	+185
		SM	28.37^A^	25.07^B^	−12	23.86^B^	−16
		RH	72.57^A^	57.22^B^	−21	57.29^B^	−21
		*T*_soil_	14.69^B^	18.71^A^	+27	20.21^A^	+38

*Mean values are represented for each cluster. The relative change (Δ%) in the mean with respect to cluster 1 is indicated for clusters 2 and 3. PPFD, photosynthetic photon flux density (μmol m^−2^ s^−1^); T_air_, air temperature (°C); VPD, vapour pressure deficit (kPa); SM, soil moisture at a depth of 5 cm (% v/v); RH, relative air humidity (%); T_soil_, temperature of soil at a depth of 2 cm (°C). Different letters indicate significant differences among the clusters (Tukey HSD, α = 0.05) within species, ecosystem, or micrometeorological data*.

**Figure 3:**
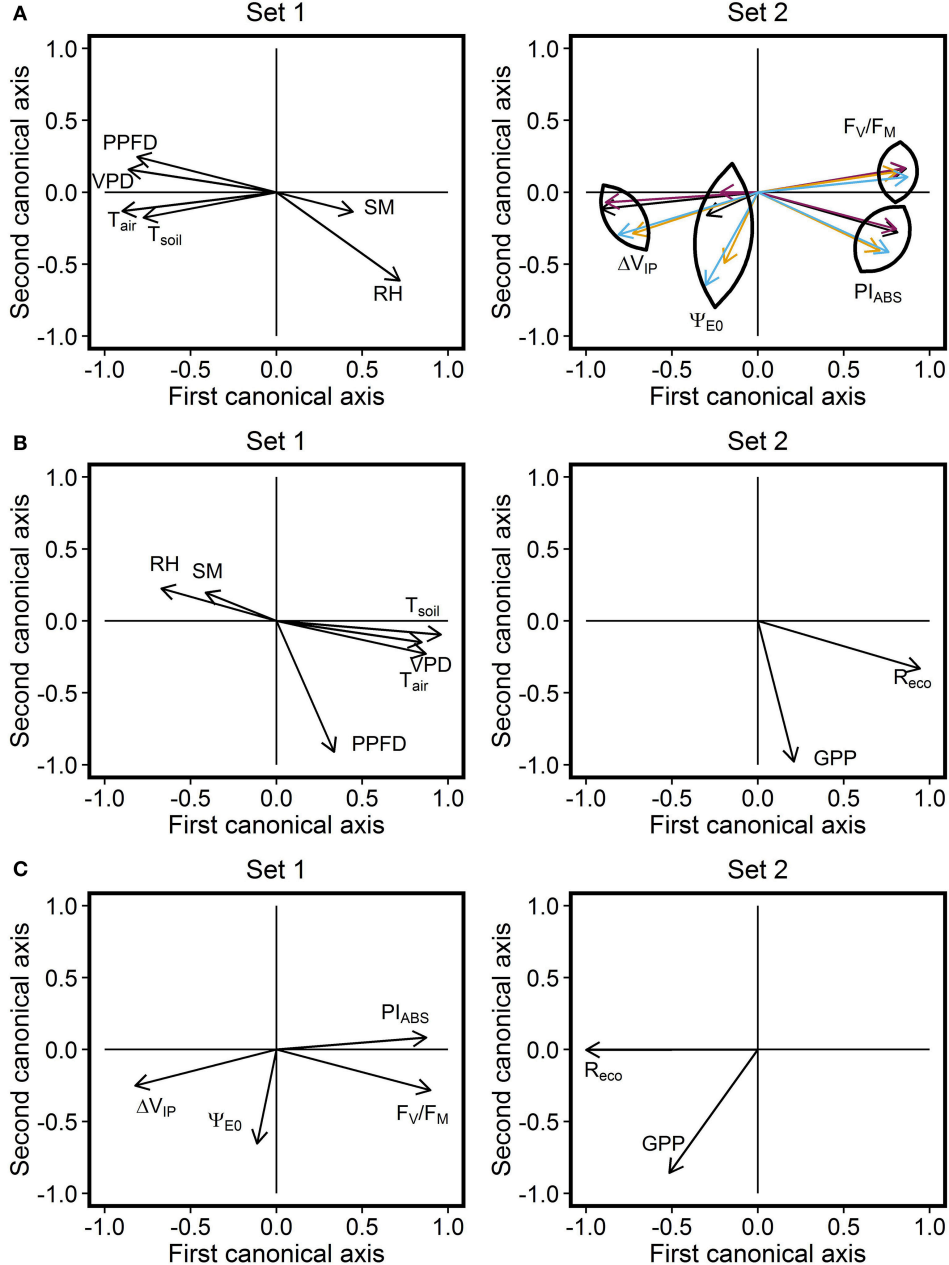
Correlation circle derived from the Canonical Correlation Analysis (CCA). CCA was performed by combining the **(A)** meteorological parameters and ChlF parameters (with distinction between purple, *L. perenne*; orange, *Taraxacum* sp.; blue, *T. repens*; black, ecosystem) data. This analysis establishes a multivariate relationship between parameters from the two datasets. Correlations between the first canonical axis of the two CCA plots and between the second canonical axis of the two CCA plots were 88.6% [Wilks' Lambda = 0.071, *F*_(96, 1, 173)_ = 7.26, *P* < 0.001] and 60.5% [Wilks' Lambda = 0.333, *F*_(75, 995)_ = 3.42, *P* < 0.001], respectively. The same approach was then used by combining **(B)** meteorological parameters and CO_2_ flux data. Correlations between the first canonical axis of the two CCA plots and between the second canonical axis of the two CCA plots were 78.8% [Wilks' Lambda = 0.266, *F*_(12, 402)_ = 31.44, *P* < 0.001] and 54.7% [Wilks' Lambda = 0.701, *F*_(5, 202)_ = 17.26, *P* < 0.001], respectively. Finally, the same approach was used by combining **(C)** ecosystem ChlF parameters and CO_2_ flux data. Correlations between the first canonical axis of the two CCA plot and between the second canonical axis of the two CCA plots were 72.4% [Wilks' Lambda = 0.468, *F*_(8, 422)_ = 24.4, *P* < 0.001] and 15.6% [Wilks' Lambda = 0.976, *F*_(3, 212)_ = 1.8, *P* = 0.155], respectively. PPFD, photosynthetic photon flux density (μmol m^−2^ s^−1^); *T*_air_, air temperature (°C); VPD, vapor pressure deficit (kPa); SM, soil moisture at a depth of 5 cm (% v/v); RH, relative air humidity (%); *T*_soil_, temperature of soil at a depth of 2 cm (°C); *R*_eco_, respiration of the ecosystem (μmol CO_2_ m^−2^ s^−1^); GPP, gross primary productivity (μmol CO_2_ m^−2^ s^−1^).

CCA analysis revealed significant correlations between environmental factors and the photosynthetic performance of the studied grassland species and the ecosystem; correlation for the first and the second canonical pairs equalled to 88.6% (*P* < 0.001) and 60.5% (*P* < 0.001) respectively (Figure 3A). ChlF parameters *F*_V_/*F*_M_, PI_ABS_, and Δ*V*_IP_ showed similar relationships with environmental parameters for the three species. Decreased *F*_V_/*F*_M_ and PI_ABS_ was associated with increased PPFD, VPD, *T*_air_, and *T*_soil_, and decreased RH and SM. These results suggested a decline in photosynthetic performance under these conditions, typically observed in summer, especially at midday. Compared with *F*_V_/*F*_M_ and PI_ABS_, Δ*V*_IP_ exhibited an opposite relationship with environmental parameters. The parameter representative of electron transport efficiency beyond Q_A_, Ψ_E0_, was least influenced by meteorological parameters, particularly for *L. perenne*. SM exhibited the lowest influence on ChlF parameters. ChlF parameters which described the ecosystem photosynthetic performance exhibited a similar relationship with meteorological parameters than ChlF parameters describing *L. perenne* response.

### Influence of environmental conditions and ChlF-based photosynthetic parameters on Co_2_ fluxes

Significant correlations between environmental factors and CO_2_ fluxes, with correlations for the first and the second canonical pairs equal to 78.8% (*P* < 0.001) and 54.7% (*P* < 0.001) respectively, were shown by the CCA (Figure 3B). Elevated *R*_eco_ and GPP fluxes were associated with high PPFD, *T*_air_, *T*_soil_, and VPD, but low RH- and SM-values. *R*_eco_ was primarily correlated with temperature and VPD, whereas GPP was mainly correlated with PPFD, suggesting an augmentation of photosynthetic activity with increased light energy availability.

The same approach was used to determine the influence of ecosystem photosynthetic performance on CO_2_ fluxes (Figure 3C). The correlation for the first canonical pair was 72.4% (*P* < 0.001) while the correlation for the second canonical pair was not significant (*P* = 0.155). The relationship between ChlF parameters and CO_2_ fluxes was therefore defined by the correlation of variables on the first canonical pair. High CO_2_ flux values were associated with high Δ*V*_IP_-values and low *F*_*V*_*/F*_*M*_- and PI_ABS_-values. This was particularly noted in *R*_eco_, where GPP was mainly represented on the second canonical axis and poorly correlated with ChlF parameter variation. The ChlF parameter Ψ_E0_ was least related with CO_2_ flux changes, as it was poorly represented on the first canonical axis.

**Figure 4:**
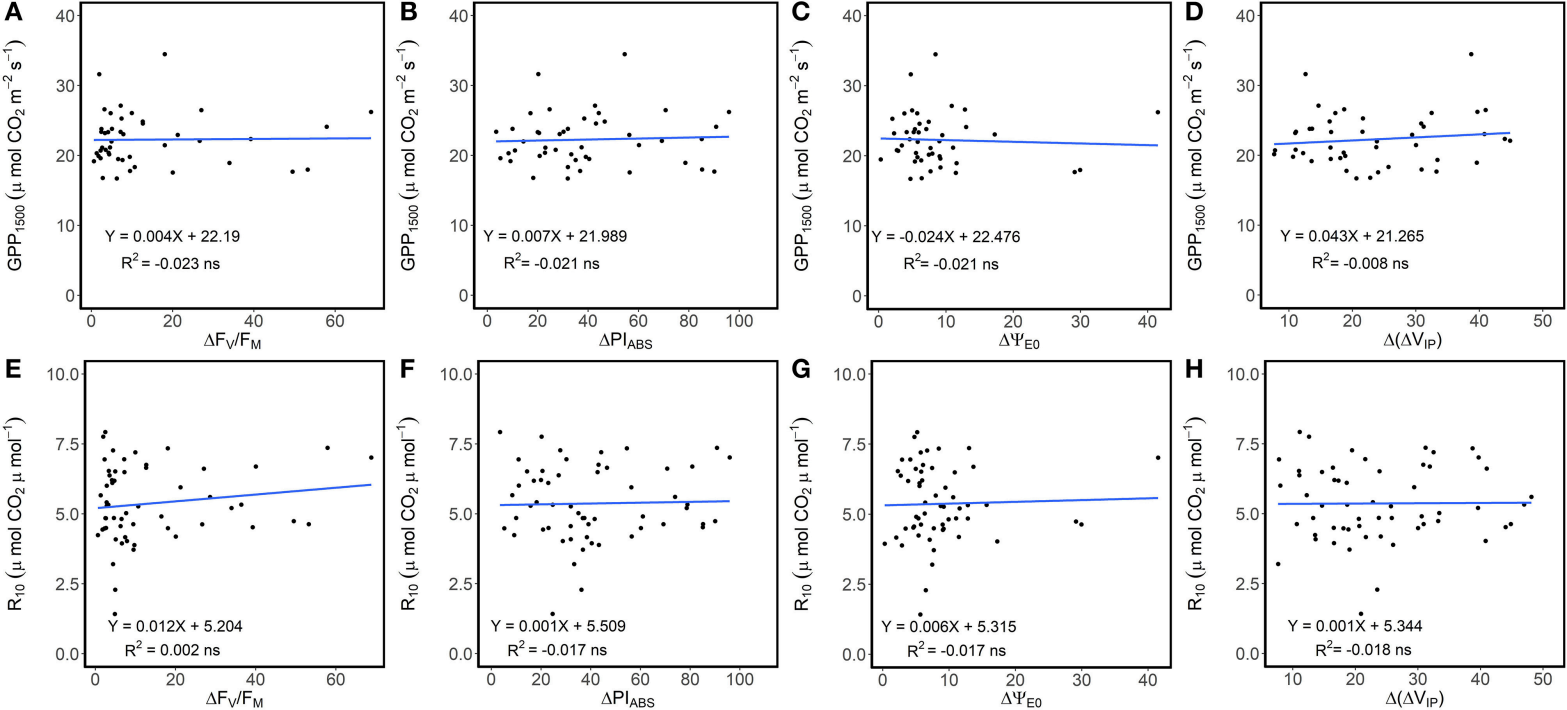
Linear regression between **(A–D)** gross primary productivity at light saturation (GPP_1500_) and the daily variation of ecosystem ChlF parameters (F_V_/F_M_, PI_ABS_, Ψ_E0_ and Δ*V*_IP_) and **(E–H)** dark respiration normalized at 10 °C (*R*_10_) and the daily variation of ecosystem ChlF parameters. Data were not root squared transformed for this test, but relationships were similar on transformed data and results are not dependent of this choice.

**Figure 5:**
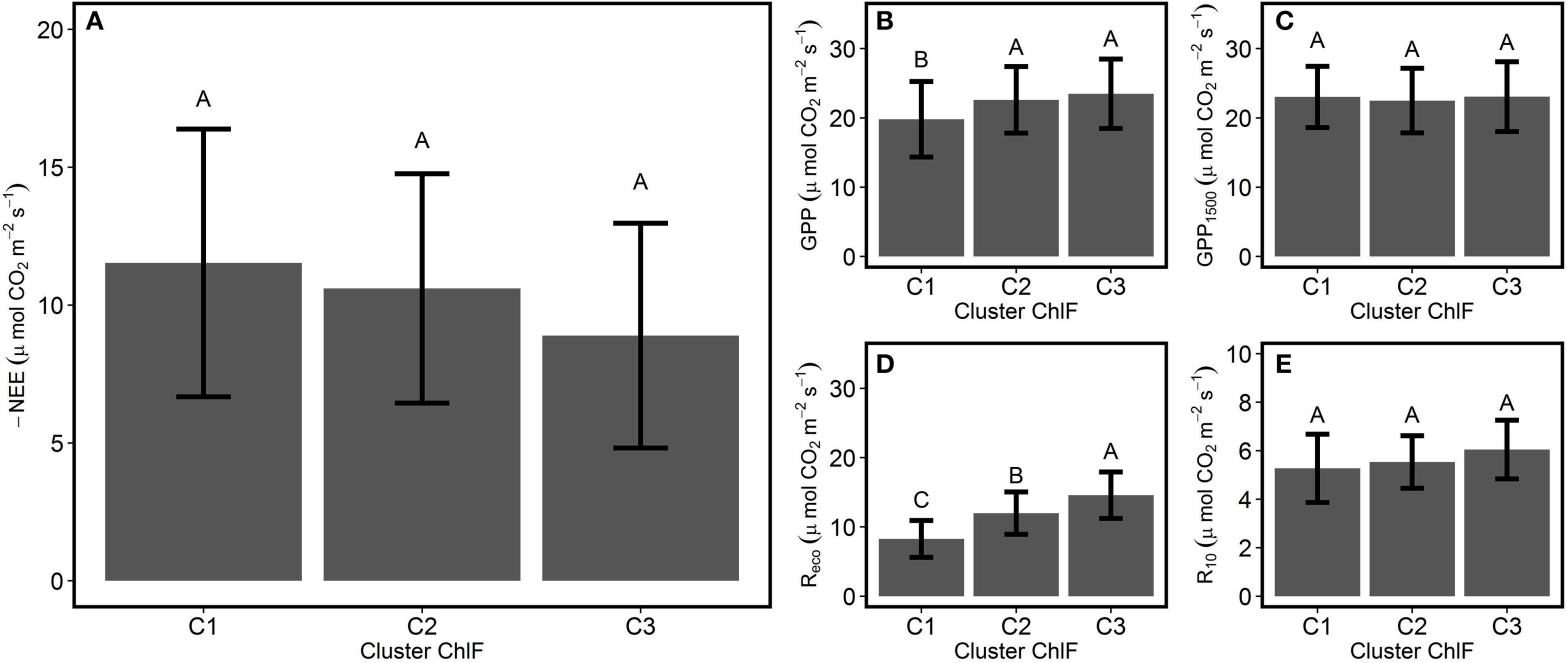
Average CO_2_ fluxes associated with the different ecosystem photosynthetic performance clusters. The average ± SD **(A)** NEE, net ecosystem CO_2_ exchange; **(B)** GPP, gross primary productivity; **(C)** GPP_1500_, GPP at light saturation **(D)** R_eco_, respiration of the ecosystem; and **(E)** *R*_10_, dark respiration normalized at 10°C were compared for the different photosynthetic performance responses based on the ChlF clusters defined during the PCA-clustering analysis. Different letters indicate significant differences among the clusters (Tukey HSD, α = 0.05).

Linear regression detected the absence of significant relationships between variation in ChlF parameters with GPP_1500_ and *R*_10_ (Figure 4), suggesting the influence of changes in photosynthetic processes was negligible. Average NEE decreased in absolute value with decreased ecosystem photosynthetic performance (Figure 5A). However, reduction in net CO_2_ uptake by the grassland was not significant. Ecosystem respiration exhibited an opposite trend and increased with decreased ecosystem photosynthetic performance (Figure 5D), probably due to its strong correlation with temperature (Figure 3B). Stable *R*_10_-values detected in the three photosynthetic performance clusters (Figure 5E) supported this hypothesis. The lowest GPP values were observed during periods of high photosynthetic performance, while the highest GPP values were observed at lower ecosystem photosynthetic performance (i.e., C2 and C3 clusters) (Figure 5B). Difference in GPP between the C2 and C3 ChlF clusters was not significant, despite reduced potential performance of photosynthesis in the C3 cluster. The three photosynthetic performance clusters showed the absence of differences in GPP_1500_-values (Figure 5C), indicating GPP_1500_ was not affected by ecosystem photosynthetic performance.

## Discussion

### Combined abiotic stresses in summer led to a decline in PSII photosynthetic performance

The three grassland species examined showed strong decreases in photosynthetic performance in summer (Figure 2), when environmental constraints were combined (Figure 1). Important *F*_V_/*F*_M_ decreases in summer were reported in various ecosystems (Fernández-Baco et al., 1998; Bussotti, 2004; Ciccarelli et al., 2016) and primarily attributed to an excess in solar radiation. A relationship between *F*_V_/*F*_M_ and PPFD was confirmed in our study by a similar diurnal evolution and strong correlation between the parameters (Figure 3A). Werner et al. (2001, 2002) interpreted the reduction in PSII photochemical efficiency as a protective mechanism, serving to preserve PSII by dissipating excess light energy as heat. The associated increase in *F*_50_ also suggests the dissociation of the antenna complex from the PSII which might contribute to the reduction of the transfer of excess energy to the RC (Mathur and Jajoo, 2015). Such reorganization within the thylakoid membrane has been observed in *Arabidopsis thaliana* (ecotype Col-0) after long-term acclimatization to high light and was characterized by the detachment of the moderately bound LHCII trimer from the PSII supercomplexes (Bielczynski et al., 2016). It is also possible that a (zeaxanthin-antheraxanthin)-dependent sustained non-photochemical quenching was operating during sunny days and contributed to the reduction of *F*_V_/*F*_M_. In such situation, some components of the non-photochemical quenching can still operate after dark-adaptation and lead to the measurement of not fully unquenched *F*_M_-values (i.e., not fully relaxed in darkness) (Adams and Demmig-Adams, 2004). However, such sustained energy dissipation mechanism is usually observed at low temperature while warm condition (such as observed at midday) was observed to efficiently relax this photoprotection process (Demmig-Adams and Adams, 2006).

High temperature can also lead to reversible conformational changes in some RC, which can no longer reduce Q_A_. These “silent RC” act as excitation energy traps, where the energy is subsequently dissipated (Bussotti, 2004; Strasser et al., 2004). This reduction in the PSII donor side efficiency can lead to decreased *F*_M_ (Mathur et al., 2011), which was observed in our study (Table 2). RC inactivation also caused *F*_V_/*F*_M_ and RC/ABS reductions (data not shown), which exhibited strong diurnal decreases and were responsible for the low midday PI_ABS_ values in summer during hot sunny days (Figure 2C). End of day increased PI_ABS_ along with reduced PPFD indicated PSII functionality was not irreversibly impaired, but rather down-regulated. PI_ABS_ reductions reaching 95% in the C3 cluster for the three grassland species (Table 2) suggest a high capacity to dissipate excess excitation energy within PSII under stressful environmental conditions.

The PSII is generally described as drought-resistant (Kalaji et al., 2016) although drought-induced damages to the PSII are observed in situation of severe drought stress (Goltsev et al., 2012) especially in sensitive variety (Ghotbi-Ravandi et al., 2014). It is difficult to assess the potential impact of the two dry spells periods observed in June and July 2015 on ChlF parameters because of the low amount of data and the implication of other environmental factors during this period, especially in July due to the heat wave. Regarding the June period, most of the ChlF measurements performed during this period belonged to the high photosynthetic performance cluster, which suggests a low/negligible impact of the relatively low soil moisture content on ChlF parameters. This is in accordance with the low influence of soil moisture observed on ChlF parameters (Figure 3A). This does not exclude, however, the influence of soil moisture in combination with other environmental factors as low soil moisture was suggested to enhance PSII sensitivity to heat stress in cottonwood (Tozzi et al., 2013) and *L. perenne* at this site (Digrado et al., 2017).

### Studied dicot species exhibited the highest capacity to increase PSI efficiency

High midday Δ*V*_IP_-values measured in summer (Figure 2E) suggested up-regulation of the photochemical pathway for de-excitation under stressful conditions (Pollastrini et al., 2011; Desotgiu et al., 2012a), particularly in dicot species (Table 2). Cascio et al. (2010) suggested rapid reduction of the end electron acceptor might play a role in protecting the photosynthetic structure by limiting the accumulation of unmanaged electrons beyond the PSI acceptor side, which contributes to the formation of ROS in sunny environments. Increased PSI efficiency was accompanied by increased electron flux efficiency beyond Q_A_ in the C3 cluster for dicots, especially in *T. repens* (Table 2). It is possible that electron flow in the electron transport chain was stimulated by increased PSI efficiency. Interestingly, these changes in Δ*V*_IP_ and Ψ_E0_ in the C3 cluster were more related to an increase in air temperature than in light availability (Table 2) emphasizing the influence of temperature in these processes. This might suggests that PSI accepted almost all electrons as electron supply by the PSII was partly inhibited by high temperature (Brestic et al., 2012).

In contrast, *L. perenne* did not exhibit increased electron transport efficiency beyond PSI between the C2 and C3 clusters and a decrease in Ψ_E0_ was observed (Table 2). This limitation in PSI efficiency could be attributed to PSI photoinhibition due to light-induced oxidation of the PSI iron-sulfur component commonly observed in condition of chilling temperature (Scheller and Haldrup, 2005). It was also shown in a recent study that such inactivation of PSI can be observed at high light and at room temperature (Tiwari et al., 2016). Inactivation of the PSI induced by light, however, would require a flux of electrons from the PSII higher than the PSI acceptor side could handle. Therefore, photoinhibition of PSI could be questioned in our case as a decrease in the efficiency of electron transport beyond Q_A_ was also observed. The decrease in Ψ_E0_ observed in our study can be interpreted as a mechanism to preserve PSI under imbalance between electrons leaving PSII and those reaching electron acceptors beyond PSI (Cascio et al., 2010; Bussotti et al., 2011). A high electron flux under sunny environments might lead to the formation of “free electrons” if the availability of end electron acceptors is insufficient. The free electrons can subsequently activate oxygen and lead to hydrogen peroxide production (Asada, 2006). Reduction in electron transport efficiency beyond Q_A_ might therefore contribute to the reduction of “free electron” accumulation, and therefore, oxidative damage. Further experimentations such as the measurements of the actual electron flow through leaf gas exchange measurements and the monitoring of PSI RC oxidation by leaf transmission changes at the 820 nm wavelength band would be required to test and verify these hypotheses.

### Decline in photosynthetic performance did not lead to a decrease in GPP

GPP variation was well-explained by environmental conditions, such as PPFD, compared with ecosystem photosynthetic performance (Figure 3). Moreover, declined ecosystem photosynthetic performance did not result in reduced carbon uptakes (Figure 5B). The absence of GPP decrease during episodes of high-energy dissipation can be explained by increased photons received during these periods. Stable GPP-values in C2 and C3 clusters (Figure 5B) also suggested the lack of stomatal limitations to carbon assimilation in the grassland during summer stress conditions.

The carbon amounts fixed by the ecosystem at light saturation were not affected by the ecosystem photosynthetic performance (Figure 5C) or variation in the different photosynthetic processes (Figure 4A). A close relationship between *F*_V_/*F*_M_ and carbon uptake was found in studies on maize plants and fodder shrubs, particularly under severe stress, when non-stomatal components effected photosynthesis (Xu et al., 2008; Boughalleb et al., 2009). Our results suggested the absence of non-stomatal limitations in photosynthesis and high ecosystem tolerance to environmental stress during the study but for a particular period of time in which high VPD and *T*_air_ were recorded.

A previous study conducted at the Dorinne Terrestrial Observatory has shown that herbage height was subjected to small variation during the grazing season as the stocking density was adapted to plants (re)growth by the farmer (Gourlez de la Motte et al., 2016). Beginning of the grazing season in May 2015, however, was characterized by a higher herbage height which might have been responsible for the higher GPP and the stronger negative NEE fluxes observed at this period (Figure 2F) as higher biomass has been associated to higher negative NEE in another grazed temperate grassland study (Zhu et al., 2015). Outside this period, we do not expect a significant influence of variation in biomass on CO_2_ fluxes as herbage height only fluctuated by around 2 cm (data not shown).

### Photosynthesis saturation was associated with PSI limitations to manage high electron flux

Results indicated a lack of GPP increase between C2 and C3 clusters despite increasing temperature, suggesting optimal temperature conditions and/or photosynthetic saturation due to high PPFD recorded under these conditions. Subsequently, an associated absence of significant increased Δ*V*_IP_ was observed for the ecosystem whereas dicot species exhibited a significant Δ*V*_IP_ increase in these conditions (Table 2). The incapacity for additional carbon assimilation by the grassland ecosystem under these conditions might be attributed to an impairment of the PSI acceptor side to manage a higher electron flux. Indeed, a high correlation between net photosynthesis and I-P phase related parameters was found in ozone stress studies under high light (Cascio et al., 2010; Desotgiu et al., 2012b), suggesting this OJIP curve region might be applied as a probe for the photosystem's capacity to feed the Calvin-Benson cycle. In our study, however, we detected a weak correlation (*r* = 0.111, *P* < 0.001, data not shown) between the I-P phase and photosynthetic activity (i.e., GPP) compared with *r*-values reported in the aforementioned studies (*r* = *0*.324 and 0.425 for Cascio et al., 2010 and Desotgiu et al., 2012b, respectively). The lower coefficient we observed might be explained because gas-exchanges were not measured on specific leaf species, but resulted from a blend of different species.

It is also likely that photorespiration activity in the grassland have increased with increasing temperature (Sage, 2013), competing with Rubisco carboxylation and thus reducing the apparent photosynthesis (i.e., “true photosynthesis” minus photorespiration). Photorespiration serves a protective role in the photosynthetic apparatus, particularly under excess light conditions, by consuming excess photon energy and preserving PSII from photodamage (Bai et al., 2008; Massacci et al., 2008). Increased photorespiration can stimulate the apparent linear electron transport, which is not fully dedicated to carbon fixation (Rho et al., 2012; Osório et al., 2013). Increase in the efficiency of electron transport beyond PSI and Q_A_ was measured in dicot species in the absence of GPP increase in the C3 cluster and can be indicative of an increase in photorespiration activity. Dicot species, however, only represented 11.4% of the population, which questions dicots contribution to CO_2_ fluxes. In addition, as explained in the material and methods section, GPP in high light condition is likely to more representative of “true photosynthesis,” which represents only carboxylation activity and would not reflect increase in Rubisco oxygenation. It is also possible that the observed increase in the electron transport efficiency for dicots results from enhanced cyclic electron transport, a mechanism involved in the protection of the photosynthetic apparatus under high light (Takahashi et al., 2009; Kalaji et al., 2012). This hypothesis is supported by the associated Δ*V*_IP_ increase which was also observed by Campos et al. (2014) in *Agave salmiana* Otto ex Salm-Dyck seedlings where the enhancement in cyclic electron flow had promoted PSI activity.

### High respiration activity was associated with low photosynthetic performance

Total ecosystem respiration increased with temperature in the grassland (Figure 3B), most likely due to increased enzyme activity. Aboveground vegetation and soil might have contributed to increased *R*_eco_. Indeed, several studies demonstrated that soil respiration (i.e., root and microbial components) responded positively to increasing temperature (Borchard et al., 2015; Hill et al., 2015; Chen et al., 2017). Belowground respiration might have been an important contributor to *R*_eco_, as soil respiration represented more than 70% of ecosystem respiration in an alpine grassland study (Ganjurjav et al., 2014). However, Hoover et al. (2016) cautions comparisons of CO_2_ soil fluxes among studies must be conducted carefully, as different plant communities were shown to influence soil respiration. The potential important soil respiration contribution to *R*_eco_ increase, however, does not exclude the potentially for increased vegetation respiration. Atkin and Tjoelker (2003) reported plant respiration responded positively to increasing temperature. Chen et al. (2016) also showed the response of carbon flux to warming was dependent on plant functional types in meadow grasslands, emphasizing the integral role of vegetation on aboveground fluxes. Our results did not determine the contribution of aboveground vegetation and soil to *R*_eco_ fluxes.

Increasing ecosystem respiration was also associated with decreasing photosynthetic performance (Figures 3C, 5D). This observation, however, might result from the common *R*_eco_ and photosynthetic processes sensitivity to temperature, as decreased photosynthetic performance was associated with increased temperature (Figure 3A). Moreover, the absence of a significant linear relationship between variation in ChlF parameters and *R*_10_ (Figure 4) suggested the *R*_eco_ response to temperature was not influenced by changes in photosynthetic processes measured by ChlF. Because photorespiratory activity was not taken into account in the partitioning, *R*_eco_ may have been underestimated, especially during hot and sunny days. Even though the absence of significant increase in the efficiency of electron transport beyond PSI and Q_A_ for the ecosystem suggests a negligible activity of alternative electron sink, this hypothesis need to be confirmed by gas-exchange measurements at leaf scale.

## Conclusions

Results of this study revealed contrasted responses of the photosynthetic apparatus in three grassland species under combined environmental stresses in summer. Because stress drivers are multiple and untangled, it is probable that the observed photosynthetic response for the different species results from the combination of different events such as energy dissipation within the antenna, dissociation of LHCII from the PSII supercomplexes, PSII RC silencing, regulation of the electron flow and change in PSI efficiency. The causes for the contrasted responses observed among measured species are yet to be identified and might also depend on plant/leaf anatomy as well as physiological factors such as the phloem loading capacity (Demmig-Adams and Adams, 2006). In a grassland study, Gielen et al. (2007) also suggested that difference in plants height among species might lead to difference in shading and, therefore, different susceptibility to midday photoinhibition between species. Results also highlighted that a decrease in the ecosystem photosynthetic performance did not result in reduced carbon fixation. We wonder if an increase in abundance of the studied dicot species might improve carbon fixation during stress episodes, as the two dicots expressed an enhanced capacity to stimulate PSI efficiency under stressful conditions. However, this response which was associated with improved efficiency in electron transport, might also indicate increased alternative electron sinks, such as photorespiration and/or cyclic electron flow. Further studies are needed to partition the relative contribution of these different electron sinks to PSI activity under stressful conditions. Better understanding of these mechanisms in response to environmental constraints and their impact on ecosystem CO_2_ fluxes might be useful in elucidating ecosystem response to climate change and might help in selecting cultivated varieties favoring carbon fixation during stress episodes.

## Author contributions

BH, MA, PD, PdJ, CA, NS, and M-LF: planned and designed the research; AD, LdlM, AB, CA, NS, and AM: performed experiments and conducted fieldwork; AD, LdlM, AB, FB, and A-CD: analyzed and interpreted the data; AD: wrote the paper; BH, MA, PD, PdJ, CA, NS, AB, FB, and M-LF: revised the paper.

### Conflict of interest statement

The authors declare that the research was conducted in the absence of any commercial or financial relationships that could be construed as a potential conflict of interest. The reviewer LF declared a past co-authorship with one of the authors FB to the handling Editor, who ensured that the process met the standards of a fair and objective review.
