# Supplementary material for: Decrease in the Photosynthetic Performance of Temperate Grassland Species Does Not Lead to a Decline in the Gross Primary Production of the Ecosystem
Source: Front Plant Sci. 2018 Feb 5;9:67. doi: 10.3389/fpls.2018.00067 (PMC5807415; doi:10.3389/fpls.2018.00067)
Supplement: Supplementary file 1 [file DataSheet1.docx]

Supplementary Material

Decrease in the photosynthetic performance of temperate grassland species does not lead to a decline in the gross primary production of the ecosystem

Anthony Digrado^1^, Louis Gourlez de la Motte^2^, Aurélie Bachy^2^, Ahsan Mozaffar^2,3^, Niels Schoon^3^, Filippo Bussotti^4^, Crist Amelynck^3,5^, Anne-Catherine Dalcq^6^, Marie-Laure Fauconnier^7^, Marc Aubinet^2^, Bernard Heinesch^2^, Patrick du Jardin^1^, Pierre Delaplace^1*^

*** Correspondence:** Pierre Delaplace: pierre.delaplace@ulg.ac.be

# Supplementary Figures and Tables

## Supplementary Figure


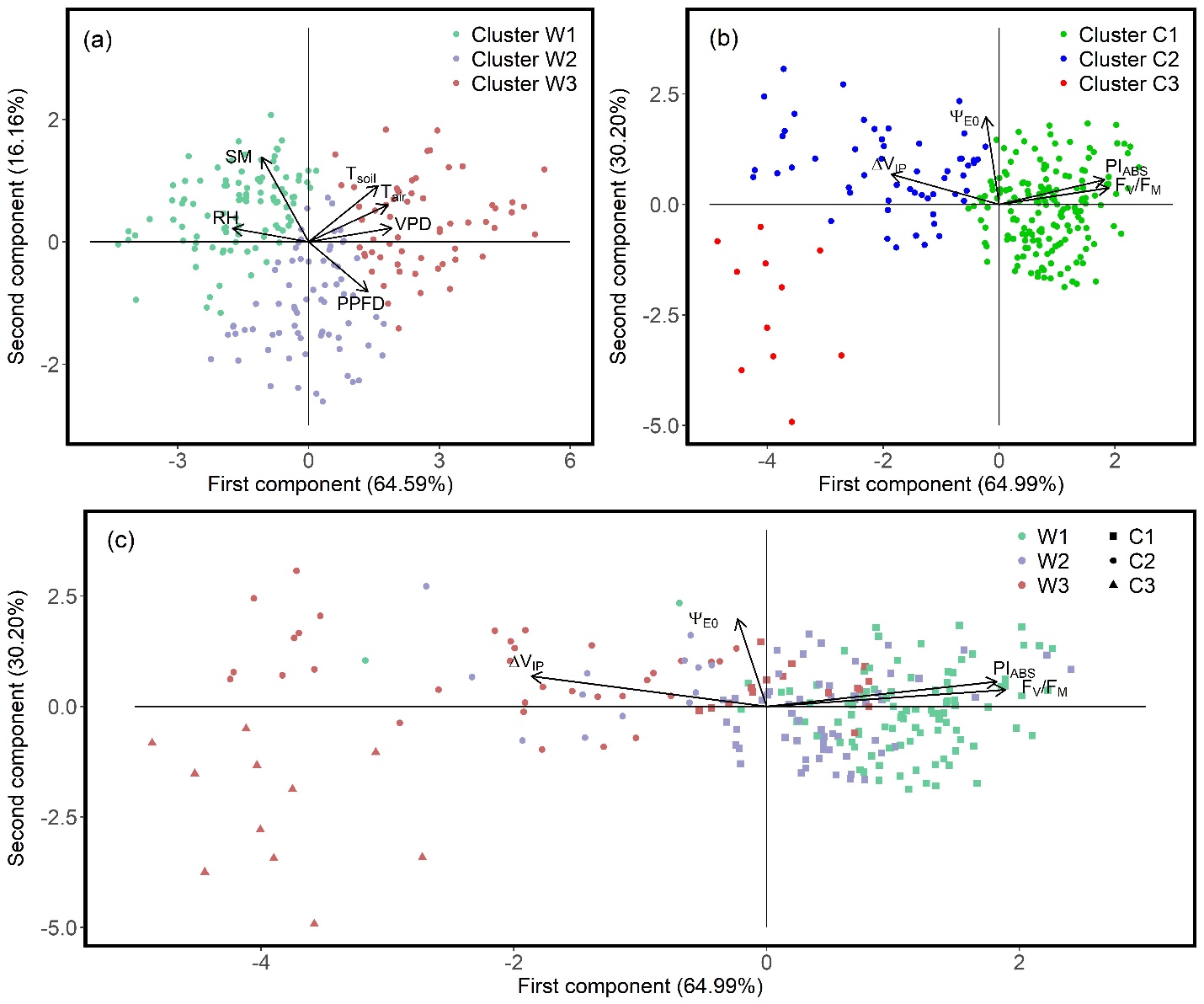


Supplementary figure 1. Results of the PCA-clustering analysis with variables. (a) Three cluster groups (W1, W2, W3) were obtained by PCA-clustering on meteorological parameters (PPFD, T_air_, SM, VPD, SM, T_soil_). The assigned cluster number for each time period of measurement was then used as a meteorological factor in a GLM analysis to test the influence of the block factor. (b) Three cluster groups (C1, C2, C3) were obtained by PCA-clustering on ChlF parameters (F_V_/F_M_, PI_ABS_, Ψ_E0_ and ΔV_IP_) estimated for the ecosystem. (c) Representation of the overlap between the meteorological condition (W1, W2, W3; colour scale) and the ChlF response (C1, C2, C3; shape scale) in the PCA environment computed based on ChlF data estimated for the ecosystem.


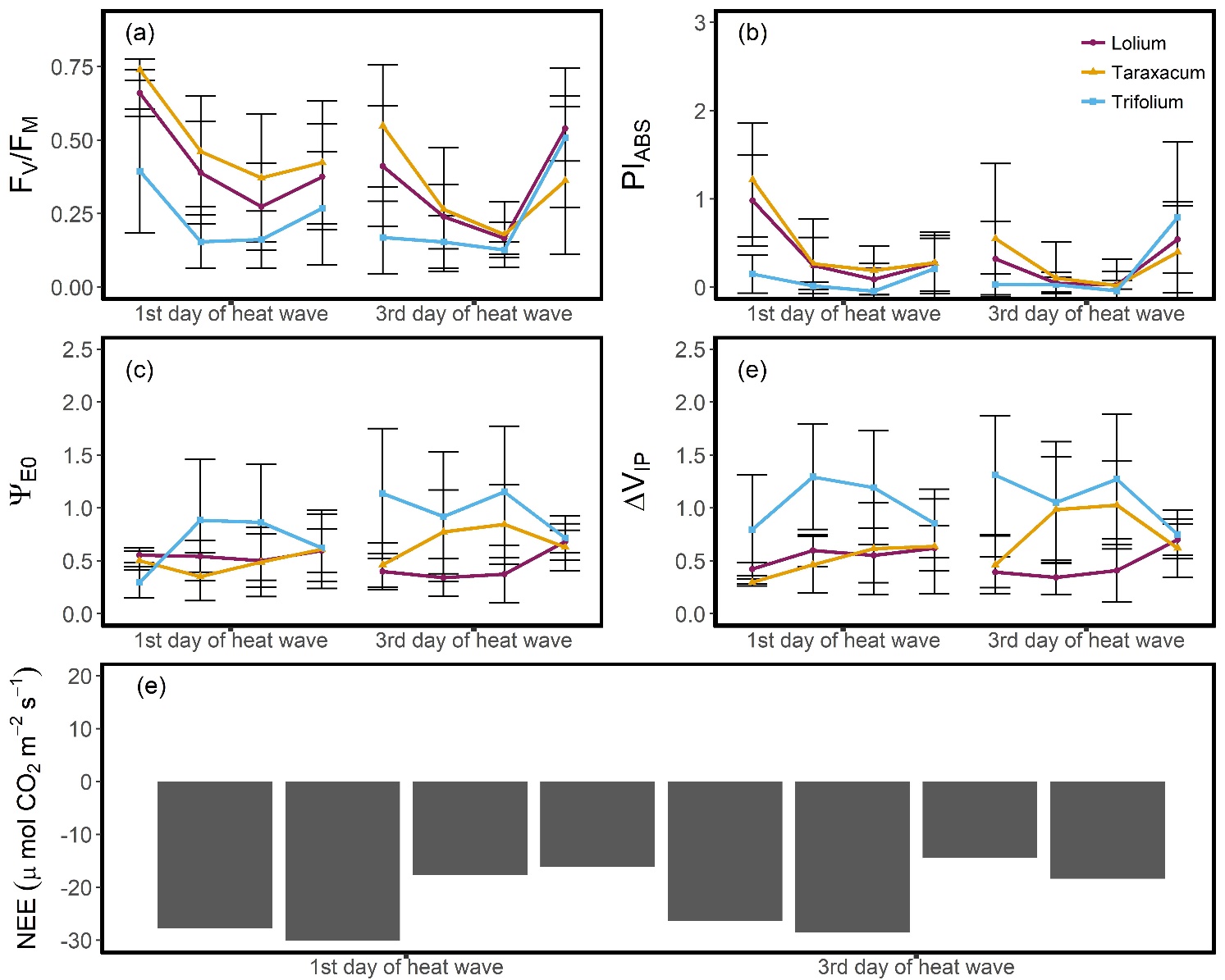


Supplementary figure 2. Time course of ChlF parameters (F_V_/F_M_, PI_ABS_, Ψ_E0_ and ΔV_IP_) for the three grassland species (purple, L. perenne; orange, Taraxacum sp.; light blue, T. repens) and net CO_2_ exchange ecosystem (NEE, µmol CO_2_ m^-2^ s^-1^) during the first and third day of a heat wave measured in the grassland. For each measured day, the ChlF parameter average value (n = 21 or 24) ± SD for each of the four measurement time periods (11:00, 13:00, 15:00, and 17:00 h) is represented for the three grassland species.

## Supplementary Table

Supplementary table 1. Botanical diversity evaluated on 24 quadrats (0.5 x 0.5 m) during September 2010 and June 2011 in the grassland. The number of quadrats where the species were identified (Presence), the frequency or probability of finding the species in a quadrat (F), the frequency relative to the total presence of all species (P), and the relative abundance (B). The relative abundance was calculated as the sum of the abundance index of the considered species for the 24 quadrats divided by the total abundance index for all species. The abundance index was based on the surface occupation of the considered species inside the quadrat and was evaluated on a scale from 0 to 3 the first year and on a scale from 0 to 10 the second year.

|  | September 2010 | | | | June 2011 | | | |
| --- | --- | --- | --- | --- | --- | --- | --- | --- |
| Species | Presence | F [%] | P [%] | B [%] | Presence | F [%] | P [%] | B [%] |
| Grass | 70 |  | 62.5 | 75.2 | 100 |  | 68.0 | 88.6 |
| *Agrostis stolonifera* L. | 13 | 54.2 | 11.6 | 6.3 | 12 | 48.0 | 8.2 | 4.2 |
| *Alopecurus geniculatus* L. | 0 | 0.0 | 0.0 | 0.0 | 9 | 36.0 | 6.1 | 2.4 |
| *Bromus hordeaceus* L. | 0 | 0.0 | 0.0 | 0.0 | 1 | 4.0 | 0.7 | 0.2 |
| *Cynosurus cristatus* L. | 5 | 20.8 | 4.5 | 4.0 | 5 | 20.0 | 3.4 | 1.2 |
| *Dactylis glomerata* L. | 6 | 25.0 | 5.4 | 3.6 | 7 | 28.0 | 4.8 | 5.0 |
| *Elymus repens* (L.) Gould | 0 | 0.0 | 0.0 | 0.0 | 3 | 12.0 | 2.0 | 0.8 |
| *Festuca pratensis* (Huds.) P. Beauv. | 2 | 8.3 | 1.8 | 2.3 | 0 | 0.0 | 0.0 | 0.0 |
| *Holcus lanatus* L. | 11 | 45.8 | 9.8 | 9.9 | 11 | 44.0 | 7.5 | 4.6 |
| *Lolium multiflorum* Lam. | 0 | 0.0 | 0.0 | 0.0 | 1 | 4.0 | 0.7 | 0.2 |
| *Lolium perenne* L. | 24 | 100.0 | 21.4 | 43.7 | 25 | 100.0 | 17.0 | 61.0 |
| *Poa annua* L. | 0 | 0.0 | 0.0 | 0.0 | 1 | 4.0 | 0.7 | 0.2 |
| *Poa pratensis* L. | 1 | 4.2 | 0.9 | 0.7 | 0 | 0.0 | 0.0 | 0.0 |
| *Poa trivialis* L. | 8 | 33.3 | 7.1 | 4.6 | 25 | 100.0 | 17.0 | 8.8 |
| N-fixing dicot | 22 |  | 19.6 | 15.9 | 20 |  | 13.6 | 5.6 |
| *Trifolium repens* L. | 22 | 91.7 | 19.6 | 15.9 | 20 | 80.0 | 13.6 | 5.6 |
| Non-N-fixing dicots | 20 |  | 17.9 | 8.9 | 27 |  | 13.6 | 5.8 |
| *Capsella bursa-pastoris* (L.) Medik. | 0 | 0.0 | 0.0 | 0.0 | 2 | 8.0 | 1.4 | 0.4 |
| *Carduus* L. | 1 | 4.2 | 0.9 | 0.3 | 2 | 8.0 | 1.4 | 0.6 |
| *Matricaria discoidea* DC. | 0 | 0.0 | 0.0 | 0.0 | 2 | 8.0 | 1.4 | 0.4 |
| *Plantago major* L. | 3 | 12.5 | 2.7 | 2.0 | 2 | 8.0 | 1.4 | 0.6 |
| *Ranunculus repens* L. | 1 | 4.2 | 0.9 | 0.3 | 6 | 24.0 | 4.1 | 1.4 |
| *Stellaria media* (L.) Vill. | 2 | 8.3 | 1.8 | 0.7 | 2 | 8.0 | 1.4 | 0.2 |
| *Taraxacum* sp. | 13 | 54.2 | 11.6 | 5.6 | 11 | 44.0 | 7.5 | 2.2 |
| Total | 112 |  |  | 100 | 147 |  |  | 100 |
